# Propofol-induced MiR-20b expression initiates endogenous cellular signal changes mitigating hypoxia/re-oxygenation-induced endothelial autophagy in vitro

**DOI:** 10.1038/s41419-020-02828-9

**Published:** 2020-08-13

**Authors:** Yue Lu, Sijie Wang, Shuyun Cai, Xiaoxia Gu, Jingjing Wang, Yue Yang, Zhe Hu, Xihe Zhang, Yongcai Ye, Siman Shen, Kiran Joshi, Daqing Ma, Liangqing Zhang

**Affiliations:** 1 Department of Anesthesiology, Affiliated Hospital of Guangdong Medical University, Zhanjiang, 524001 China; 2 Clinical Research Center, Affiliated Hospital of Guangdong Medical University, Zhanjiang, 524001 China; 3 Department of Anesthesiology, Xiangya Hospital, Central South University, Changsha, 410000 China; 4 Division of Anaesthetics, Pain Medicine and Intensive Care, Department of Surgery and Cancer, Faculty of Medicine, Imperial College London, Chelsea and Westminster Hospital, London, UK

**Keywords:** Autophagy, Cell signalling, Genetics research

## Abstract

Certain miRNAs can attenuate hypoxia/re-oxygenation-induced autophagic cell death reported in our previous studies, but how these miRNAs regulate the autophagy-related cellular signaling pathway in preventing cell death is largely unknown. In the current study, the autophagy-related miRNAs of hsa-miR-20b were investigated in an in vitro model of hypoxia/re-oxygenation-induced endothelial autophagic cell death. Of these, miR-20b was found to be the most important miRNA which targeted on the key autophagy kinase ULK1 and inhibited hypoxia/re-oxygenation injury-induced autophagy by decreasing both autophagosomes and LC3I to II transition rate and P62 degradation. These processes were reversed by the transfection of an miR-20b inhibitor. Re-expression of ULK1 restores miR-20b-inhibited autophagy. Propofol, a commonly used anesthetic, promoted miR-20b and METTL3 expression and attenuated endothelial autophagic cell death. The inhibited endogenous expression of miR-20b or silenced METTL3 diminished the protective effect of propofol and accentuated autophagy. Additionally, METTL3 knockdown significantly inhibited miR-20b expression but up-regulated pri-miR-20b expression. Together, our data shows that propofol protects against endothelial autophagic cell death induced by hypoxia/re-oxygenation injury, associated with activation of METTL3/miR-20b/ULK1 cellular signaling.

## Introduction

Endothelial cell functional and structural damage together with other risk factors including adhesion molecules, inflammation cytokines, and clotting factors^1–4^ are the underlying causes of vital organ injury or cardiovascular disease. These pathological changes finally result in the development of vascular obstruction with angiosclerosis, thrombogenesis, and atheromatous plaque formation to block blood supply towards stroke and/or myocardial infarction^5^. On the other hand, reperfusion following thrombolytic therapy or stent implantation can cause further vital organ cell injury including endothelial cell injury. Thus, how to effectively protect from organ and endothelial cell injury induced by any insults including ischemia reperfusion is the key of the prevention and/or treatment of vital organ injury or cardiovascular disease.

Autophagy is a physiological process in which damaged proteins or cell organelles are sequestered within autophagosomes and then fuse with lysosomes for degradation^6^. However, excessive autophagy can promote cell death, especially in myocardial cells and endothelial cells. As the core component of the autophagy initiation complex, ULK1 plays an important role in autophagy^7^. Environmental factors such as a lack of nutrition, viral infection, or insulting conditions, may stimulate ULK1 and Beclin1 expression or over-expression which in turn may lead to cell death^8,9^. Early studies suggest that myocardial cell survival in stressful environments can be improved significantly through autophagy activation^10,11^. However, dysregulated and sustained autophagy in certain circumstances can inhibit cell proliferation, and even accelerate myocardial cell death^12,13^. Reperfusion injury can be the consequence of immoderate autophagy, but the motive of its duration during reperfusion injury is still largely unknown^14^. Therefore, the direct contribution of the key factor ULK1 in the process of ischemia reperfusion injury requires further study.

Propofol, a widely used intravenous anesthetic, has been reported to inhibit ROS-mediated lipid peroxidation, alleviating myocardial reperfusion injury and reducing myocardial stress in patients undergoing cardiac surgery^15^. Studies have found that propofol inhibits the activity of nuclear transcription factor NF-kβ during ischemia reperfusion injury and reduces the adhesion force of endotheliocytes^16^. In addition, propofol inhibits hydrogen peroxide-induced myocardial apoptosis and attenuates myocardial ischemia reperfusion injury by regulating autophagy-related genes^17–21^.

MicroRNAs (miRNAs), a novel class of short and non-coding RNAs which contain 20–24 nucleotides, are intracellular post-transcriptional gene regulators that negatively control gene expression via degradation or translation inhibition of their target mRNAs^22^. A previous study demonstrated that the high expression of miR-204 in myocardial cells can significantly suppress the cardiomyocyte autophagy induced by ischemia reperfusion injury, as miR-204 can inhibit the expression of LC3II^23^. Recently, it has been shown that propofol post-treatment in an in vitro model of hypoxia/re-oxygenation (H/R) led to a noticeable change in a series of miRNA expression^24^. However, whether there was an association between these miRNAs and the protective effect of propofol on H/R injury is still unknown.

N6-methyladenosine (m6A) RNA methylation is one of the most common transcriptional modifications, which plays a pivotal role in all stages of the life cycle of RNAs, including splicing process, stability, translation efficiency, and nuclear retention of mRNAs and noncoding RNAs^25,26^. The m6A methyltransferase complex, which contains methyltransferase like 3 (METTL3), methyltransferase like 14 (METTL14), and WT1-associated protein (WTAP), plays an important role in the modification of m6A^27,28^. Among them, METTL3 is the most reported m6A methyltransferase that can promote the maturation of miRNAs^29^.

In the present study, we found that the level of miR-20b and METTL3 were significantly increased while ULK1 was substantially reduced in the presence of propofol post-hypoxia treatment in an in vitro endothelial cell H/R model. Our current study aims to investigate the role of miR-20b, METTL3, and ULK1 on the protective effects of propofol-posttreatment against endothelial cell injury induced by hypoxia reperfusion in vitro.

## Results

### miR-20b regulated the expression of ULK1

**Fig. 1:**
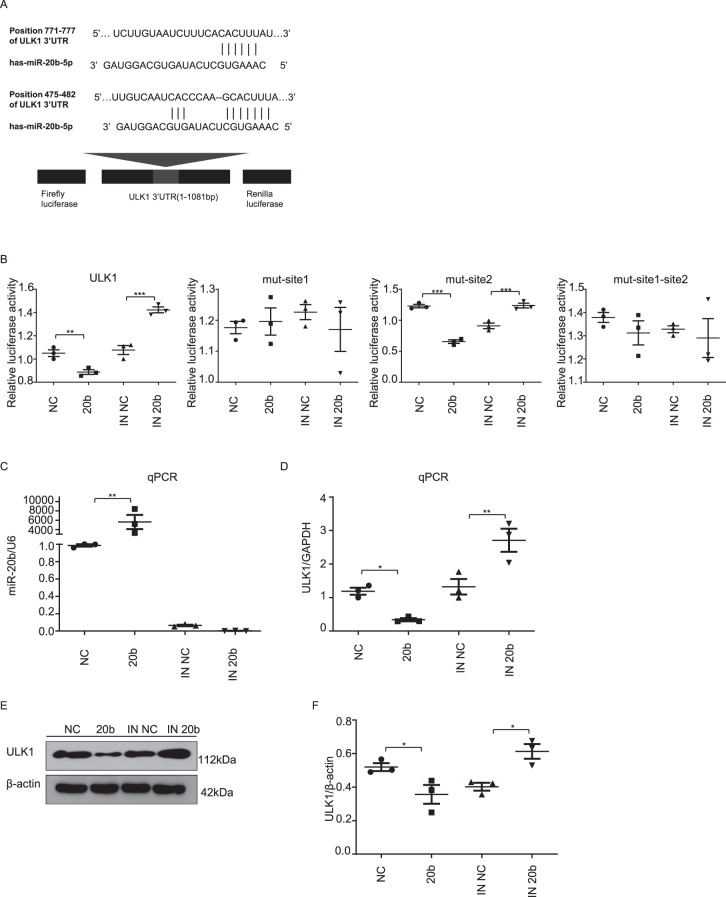
miR-20b targets ULK1 and regulates its expression. **a** Luciferase reporter constructs. The 3′UTR (1–1801 bp) of ULK1 was inserted downstream of the firefly luciferase gene of the pmirGLO vector, named pmirGLO-ULK1. MRE denotes miRNA response element. **b** Luciferase reporter assay of the interaction between miR-20b and the predicted MRE in HUVECs. Each pmirGLO-ULK1 was co-transfected with negative control (NC), miR-20b mimics (20b), inhibitor NC (IN NC), or miR-20b inhibitor (IN 20b) into HUVECs. Detection of the luciferase activity was done after 24 h. The firefly luciferase activity was normalized to Renilla. **c**, **d** qPCR was performed to detect the expression of miR-20b and ULK1 in HUVECs, respectively. **e**, **f** HUVECs lysates were prepared and subjected to western blot analysis by using anti-ULK1 antibody. Data were mean ± SEM (*n* = 3); *p < 0.05,**p < 0.01, ***p < 0.001.

ULK1 is an autophagy initiating factor that is required for autophagy induction^30,31^. MiR-20b is predicted to target ULK1 which contains two sites within the 3′UTR at nucleotides 475–482 (site 1) and 771–777 (site 2), respectively (Fig. 1a). Using a luciferase reporter construct, we showed that ULK1 activity was suppressed by miR-20b (20b) and subsequently restored through use of an miR-20b inhibitor (IN 20b) (Fig. 1b). Simultaneous mutation of both sites or only the site 1 hardly influenced the luciferase activities, but mutation of the site 2 affected the luciferase activities (Fig. 1b), indicating that site 1 is the specific binding site of miR-20b. Site-directed mutagenesis indicates that site 1 is the specific binding site for miR-20b. Luciferase activity remains constant in the presence of a site 1 mutation, but remains variable when only a site 2 mutation is present.

The expression of miR-20b and ULK1 in HUVECs was significantly decreased by the miR-20b inhibitor (IN 20b) or miR-20b mimics (20b), respectively (Fig. 1c, d). This inhibition was further verified through Western blot analysis (Fig. 1e, f). Conversely, IN 20b increased ULK1 expression through its inhibition of endogenous miR-20b.

### Propofol post-treatment inhibited on autophagy induced by H/R

**Fig. 2:**
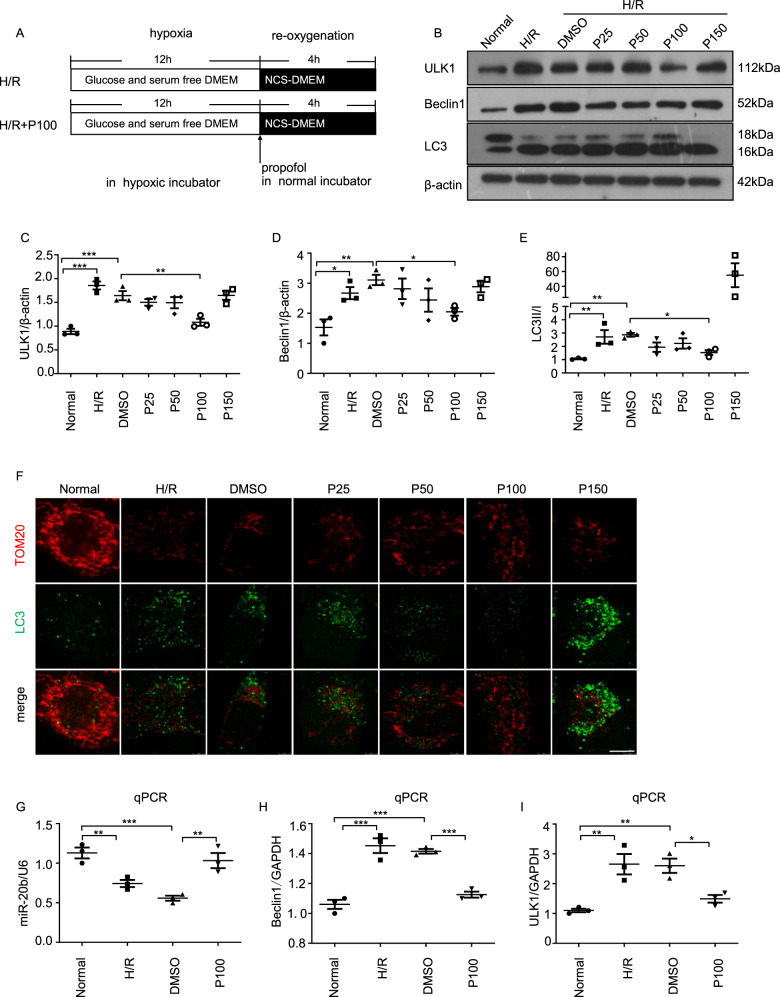
Propofol suppresses H/R-induced autophagy. **a** Built of the hypoxia/re-oxygenation model. The culture media was replaced with glucose and serum free DMEM. Then the HUVECs in hypoxic conditions was placed with 94% N_2_, 5% CO_2_, and 1% O_2_ using a small enclosed chamber filled at 37 °C for 12 h. Then the medium was changed to culture media and propofol was added with different concentrations: 25 μmol/L (P25), 50 μmol/L (P50), 100 μmol/L (P100), 150 μmol/L (P150) for 4 h in normal condition. **b**–**e** The expression of ULK1, Beclin1, and LC3 was determined in normal HUVECs, H/R injury HUVECs, H/R+DMSO HUVECs, and propofol post-hypoxia treatment HUVECs. **f** Autophagosomes and mitochondria were probed by anti-LC3 (green) and anti-TOM20 (red) in each group as above. Bar, 10 μm. Data were from three independent experiments. **g**–**i** PCR was performed to detect the expression of miR-20b, Beclin1, and ULK1 in normal HUVECs, H/R injury HUVECs, H/R+DMSO HUVECs, and propofol-treated HUVECs with the most effective concentration of 100 μmol/L. Data were mean ± SEM (*n* = 3); *p < 0.05, **p < 0.01, ***p < 0.001.

To investigate the protective effects of propofol against H/R-induced injury, cultured HUVECs were treated with propofol at various concentrations after H/R insult (Fig. 2a). H/R induced the expression of the autophagy-related protein ULK1 and Beclin1, and increased the transition of LC3I to LC3II. Those changes induced by H/R were significantly reversed by treatment with propofol, in particular at the concentration of 100 μmol/L (P100) but not of 150 μmol/L (P150) (Fig. 2b–e). Immunofluorescence staining with anti-LC3 (green) and anti-TOM20 (red) antibodies was in line with the results from Western blotting (Fig. 2f). H/R induced a significant number of LC3 puncta and fragmented mitochondria while cells were recovered to relative normal morphology upon propofol treatment except at the concentration of 150 μmol/L (*p* < 0.05). The propofol (100 μmol/L) treatment significantly reduced the expression of autophagy-related genes Beclin1 and ULK1, while inducing levels of miR-20b expression comparable to that of the H/R and DMSO group (Fig. 2g–i).

### miR-20b regulates H/R-induced autophagy by targeting ULK1

**Fig. 3:**
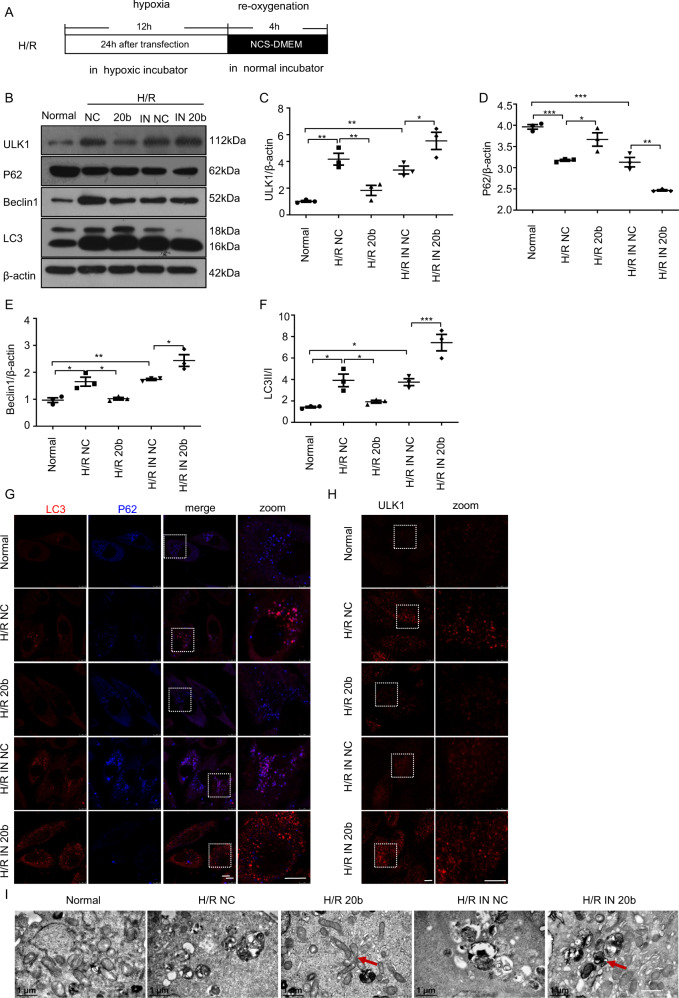
miR-20b regulates H/R-induced autophagy by targeting ULK1. **a** The HUVECs were transfected with negative control (NC), miR-20b mimics (20b), inhibitor NC (IN NC), miR-20b inhibitor (IN 20b) for 24 h. Then the HUVECs in hypoxic conditions were placed as above for 12 h. The medium was only replaced and placed in normal condition for 4 h after hypoxia. **b**–**f** Total proteins were extracted to detect ULK1, P62, Beclin1, LC3II/I (16KD/18KD) by using β-actin as a reference. Densitometric ratios of these proteins were quantified by using IMAGEJ. **g** Samples were stained with anti-LC3 (red) and anti-p62 (blue) antibodies. Bar, 10 μm. **h** Samples were stained with ULK1 (red) antibodies. Bar, 10 μm. **i** Electron microscopy was used to assess the inhibitory role of miR-20b in the H/R-induced autophagy. Bar, 1 μm. Data are mean ± SEM (*n* = 3); *p < 0.05, **p < 0.01, ***p < 0.001.

In order to verify whether miR-20b is involved in the protective effects of propofol on H/R-induced autophagy, we built the model as shown in Fig. 3a. We first investigated whether overexpression of miR-20b could restrain H/R induced-autophagy. The miR-20b-transfected HUVECs showed a high level of p62 and low ratio of LC3II/LC3I and low expression of autophagy-related proteins ULK1 and Beclin1 than that of the NC group. However, the IN 20b-transfected cells showed the opposite effect (Fig.3b–f). Similar findings were observed through immunofluorescent staining with anti-LC3 (red) and anti-P62 (blue) antibodies. MiR-20b inhibited the LC3 puncta formation while inhibiting the degradation of P62. Conversely, this effect was reversed by IN 20b (Fig. 3g). The number of ULK1 puncta (red) induced by H/R decreased upon miR-20b transfection (Fig. 3h). Electron microscopy results showed that, unlike the NC, miR-20b significantly inhibited the formation of autophagosomes under H/R condition (Fig. 3i).

### Re-expression of ULK1 restores autophagy inhibited by miR-20b

**Fig. 4:**
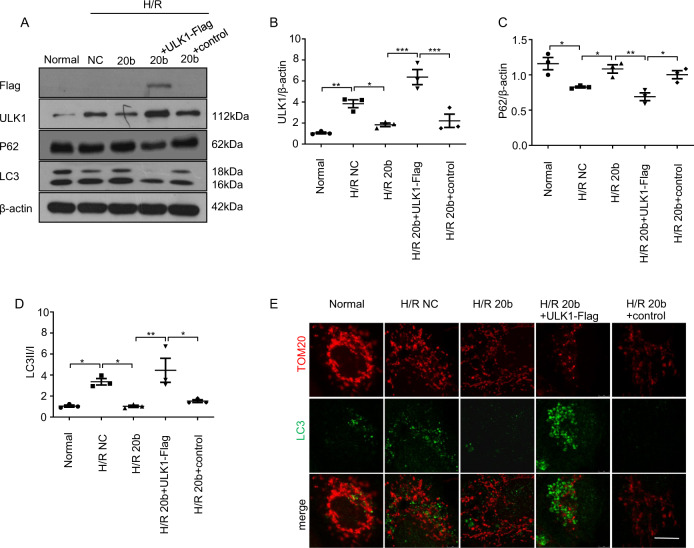
Re-expression of ULK1 restores autophagy inhibited by miR-20b. Built the H/R model as above. **a–d** HUVECs were transfected with negative control (NC) and miR-20b mimics (20b) or co-transfected with miR-20b mimics (20b) and the constructs (ULK1-Flag) were indicated for 24 h. Using a normal group as a control. Built the H/R model with these cells. Protein samples were collected and the expression of ULK1, Flag, LC3 (16KD/18KD), and P62 was detected through western blot. β-actin was used as a reference. **e** Treat the cells as in **a**. Then the cells were processed for immunofluorescence with anti-TOM20 (red) and LC3 (green) antibodies. Bar, 10 μm. Data are mean ± SEM (*n* = 3); *p < 0.05, **p < 0.01,***p < 0.001.

To verify that the autophagy-related effects of miR-20b were caused by the repression of ULK1, we evaluated whether the overexpression of ULK1-Flag, which lacks the miRNA-responsive element is resistant to miR-20b-mediated autophagy suppression. The ULK1-Flag transfection significantly induced autophagy even in the presence of miR-20b compared to those miR-20b-transfected cells (Fig. 4a). Western blot demonstrated that the H/R + miR-20b+ULK1-Flag-transfected cells (H/R 20b+ULK1-Flag) showed a significant increase in exogenous ULK1, conversion of LC3I to II, as well as the low expression of P62 when compared with miR-20b-transfected cells (H/R 20b) (Fig. 4b–d). Similarly, the immunofluorescence results were consistent with the Western blot data. The number of LC3 dots and fragmented mitochondria were significantly increased in the H/R 20b+ULK1-Flag group compared to that of the H/R 20b group (Fig. 4e).

### Inhibiting the endogenous miR-20b expression can significantly reduce the inhibitory effect of propofol on ULK1 and autophagy

**Fig. 5:**
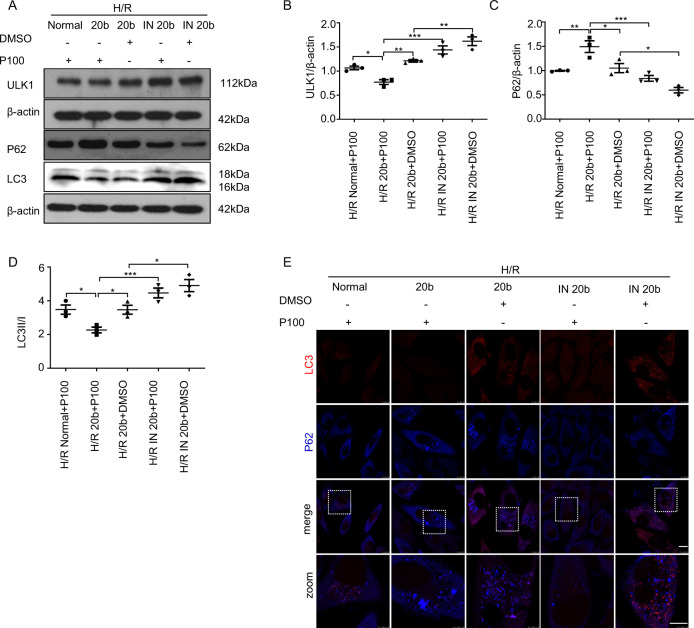
Inhibiting the expression of miR-20b reduces the inhibitory effect of propofol on autophagy. Built the H/R model as above. **a**–**d** HUVECs were transfected with miR-20b mimics (20b) or miR-20b inhibitor (IN 20b) for 24 h. Built the H/R model with these cells and the cells were treated with or without propofol at re-oxygenation for 4 h. Divided the cells into five groups: H/R Normal+P100, H/R 20b+P100, H/R 20b+DMSO, H/R IN 20b+P100, and H/R IN 20b+DMSO. Protein samples were collected and the expression of ULK1, LC3 (16KD/18KD), and P62 was detected through western blot. **e** Treat the cells as in **a**. Then the cells were processed for immunofluorescence with P62 (blue) and LC3 (red) antibodies. Bar, 10 μm. Data were mean ± SEM (*n* = 3); *p < 0.05, **p < 0.01, ***p < 0.001.

In order to confirm whether the inhibitory effect of propofol on autophagy induced by H/R was due to the existence of miR-20b, we hypothesized that the protective effects of propofol would be attenuated after suppressing endogenous miR-20b. Indeed, propofol inhibited the expression of ULK1 and the conversion of LC3 I to LC3 II, as well as degradation of P62 in the presence of miR-20b (H/R 20b + P100). Nevertheless, the autophagy-inhibiting effect of propofol was suppressed by inhibiting miR-20b expression shown in both immunofluorescence studies and Western blotting (Fig. 5a–e).

### Propofol promotes METTL3-mediated pri-miR-20b maturation

**Fig. 6:**
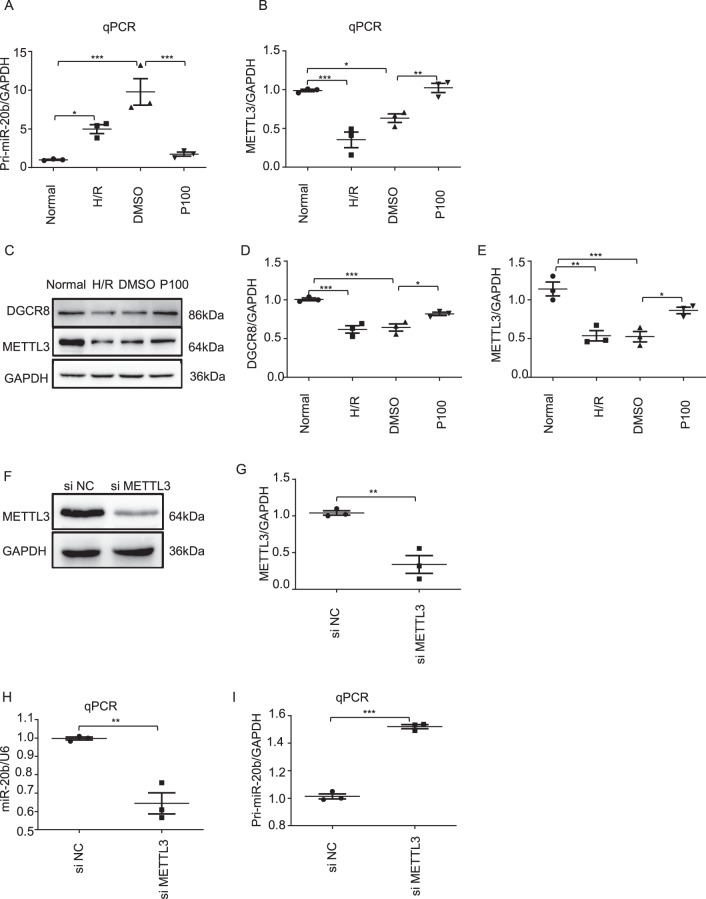
Propofol promotes METTL3-mediated pri-miR-20b maturation. Built the H/R model as above. **a**, **b** qPCR analyses of the level of pri-miR-20b and METTL3. **c**–**e** Western blotting analyses of DGCR8 and METTL3 levels. **f**, **g** Western blotting verified the successful knockdown of METTL3. **h**, **i** qPCR analysis of the level of pri-miR-20b and miR-20b after knockdown of METTL3. Data are shown as the mean ± SEM (*n* = 3); *p < 0.05, **p < 0.01, ***p < 0.001.

To elucidate the mechanisms underpinning propofol’s effects on miR-20b induction, we established the H/R model as shown in Fig. 2a and analyzed the expression levels of pri-miR-20b and METTL3. The pri-miR-20b level was significantly decreased while the METTL3 level was prominently increased in the propofol post-treatment group (P100) compared with the DMSO control (Fig. 6a, b). Furthermore, METTL3 and DGCR8 were significantly upregulated in the P100 group, compared with the DMSO group (Fig. 6c–e). METTL3 knockdown remarkably reduced the miR-20b level but increased the pri-miR-20b level (Fig. 6f–i).

### Knockdown of METTL3 restores autophagy inhibited by propofol

**Fig. 7:**
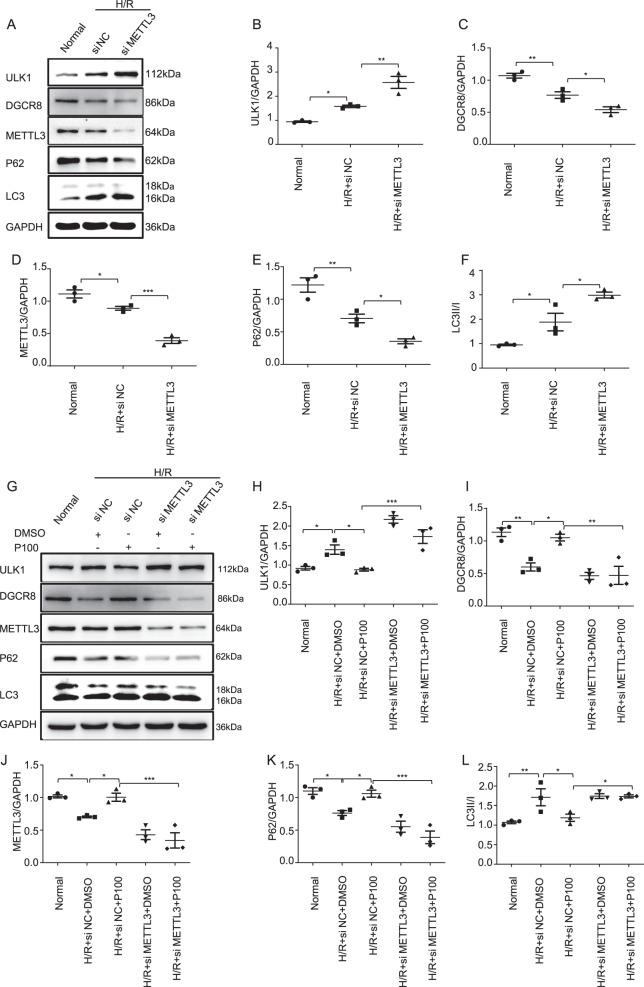
METTL3 knockdown restores autophagy inhibited by propofol. **a**–**f** Built the H/R model after knockdown of METTL3. Western blotting analysis of the autophagy-related protein and DGCR8 levels. **g**–**l** Built the H/R model after knockdown of METTL3 and treated the cells with or without propofol at re-oxygenation for 4 h. Western blotting analysis of the autophagy-related protein and DGCR8 levels. Data are shown as the mean ± SEM (*n* = 3); *p < 0.05, **p < 0.01, ***p < 0.001.

Finally, we went to explore whether METTL3 can affect autophagy inhibited by propofol. In the H/R injury model, knockdown of METTL3 leads to increased autophagy. The level of ULK1 and the ratio of LC3II/I were significantly increased, while the levels of DGCR8, P62, and METTL3 were decreased with the METTL3 knockdown (Fig. 7a–f). Moreover, the knockdown of METTL3 eliminated the protective effect of propofol in H/R injury. The expression of ULK1, DGCR8, P62, and the ratio of LC3II/I showed no difference after METTL3 knock down (H/R + si METTL3 + DMSO vs. H/R + si METTL3 + propofol) (Fig. 7g–l).

## Discussion

Our results suggest that propofol increased METTL3 expression in hypoxia reperfusion-induced endothelial injured cells. METTL3 can inhibit H/R-induced autophagy by accelerating the maturation of pri-miR-20b in an m6A-dependent manner, resulting in the reduction of ULK1. In our study, the autophagy-related protein P62 was downregulated but the level of ULK1 and the ratio of LC3II/I were upregulated under conditions of hypoxia re-oxygenation. These processes were negated by propofol given during the post-injury phase. Furthermore, propofol treatment also alleviated H/R-induced autophagy and increased the expression of miR-20b. Therefore, it can be concluded that propofol treatment inhibited endothelial cell autophagy induced by H/R injury that was likely due to an increase of the miR-20b expression and a decrease of the ULK1 expression. Our data also indicated that miR-20b is a novel autophagy-related miRNA and ULK1 was identified as a target of miR-20b, as shown by a bioinformatics analysis. MiR-20b inhibited the expression of autophagy-related proteins that were induced by H/R injury via ULK1, whereas the ULK1 overexpression in the presence of miR-20b reversed autophagy.

**Fig. 8:**
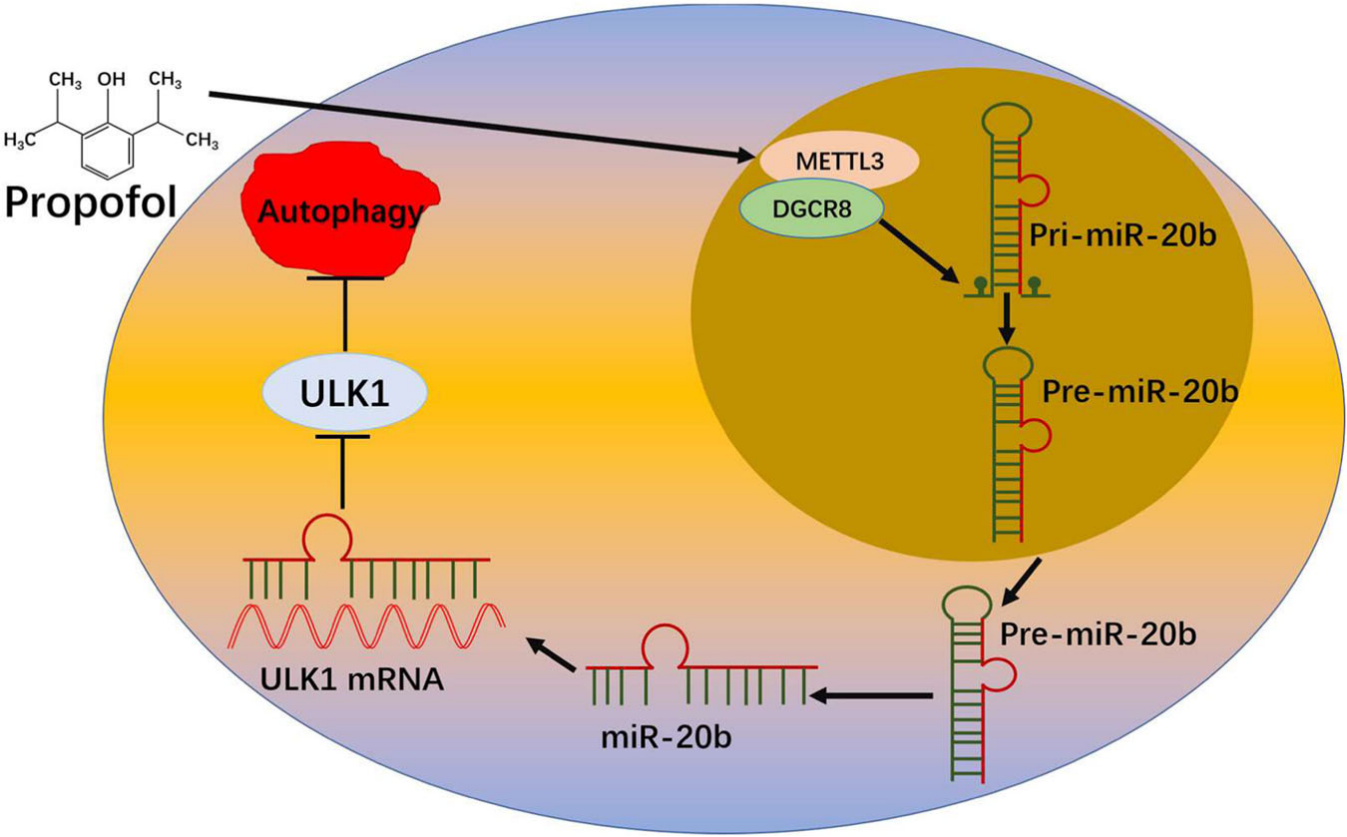
Propofol promotes cytoprotective mechanisms via miR-20b. miR-20b expression induced by propofol promotes endogenous cytoprotective mechanisms of the METTL3/miR-20b/ULK1 regulatory “network” in hypoxia/re-oxygenation injury. METTL3: methyltransferase-like 3; DGCR8: DiGeorge critical region 8; ULK1: Unc-51-like kinase 1; mRNA: Messenger RNA; pri- miR-20b: primary microRNA-20b; pre-miR-20b: precursor microRNA-20b; miR-20b: microRNA-20b.

Propofol postconditioning with miR-20b overexpression inhibited LC3 dot formation, LC3I to LC3II conversion and ULK1 production. The endogenous miR-20b suppression significantly decreased the inhibitory effect of propofol on ULK1 and autophagy. Propofol postconditioning also attenuated ULK1 and pri-miR-20b levels of the H/R-damaged cells but upregulated METTL3 and miR-20b. METTL3 knockdown significantly inhibited the expression of miR-20b but surprisingly upregulated the expression of pri-miR-20b, indicating that maturation of miR-20b from pri-miR-20b depends on METTL3. More importantly, we found that knockdown of METTL3 induced low levels of DGCR8. It may be the case that METTL3 enhances the recognition of pri-miR-20b by DGCR8 and the subsequent processing to mature miR-20b in an m6A-dependent manner. Knockdown of METTL3 significantly increased H/R-induced autophagy and mitigated the protective effects of propofol. Taken together, it can be concluded that propofol postconditioning prevents H/R-induced autophagic cell death via the METTL3/miR-20b/ULK1 signaling pathway. Our data indicates that miR-20b promotes endogenous cytoprotective mechanisms (Fig. 8). This is consistent with the results of the other studies published previously. Indeed, an in vivo model demonstrated that miR-20b promoted ventricular remodeling following myocardial IR injury in rats by inhibiting the expression of Smad7^32^. The high expression of miR-20b promoted endothelial cell viability and reduced H_2_O_2_‑induced cell senescence^33^. Another study showed that miR-20b-enriched exosomes protected against kidney stone-induced injury by suppressing autophagy and inflammatory responses^34^. As an initiator of autophagy, ULK1 regulates autophagy through interaction with upstream AMPK and mTOR cellular signals, and then transduces signals to downstream mediators^35–37^. The presence of miRNAs including miR-20b under stress conditions leads to a reduction in ULK1 and other autophagy-related protein levels below the threshold, and therefore results in a decrease in LC3 transition and inhibition of autophagic activity. Specific stimuli promoting autophagy seem to be counteracted by inhibitor miRNAs such as miR-20b, limiting excessive and potentially harmful autophagic activity in cells. Therefore, this current work may indicate that miR-20b is a new autophagy-related miRNA that works by suppressing autophagy, which may provide novel insight into the exploration of the connection of miR-20b and autophagy.

M6A is a prevalent internal modification of mRNAs, accounting for about 50% of total methylated ribonucleotides and 0.1–0.4% of all adenosines in total cellular RNAs. As one of the most common RNA modifications, m6A is found in almost all types of RNAs, and it has been implicated in a variety of cellular processes including mRNA metabolism and miRNA biogenesis^38^. METTL3, as the most important component of the “writer” complex, is known to be involved in all stages of the life cycle of RNA. It plays a pivotal role in pre-mRNA splicing^39^ microRNA (miRNA) processing^29^, 3′-end processing^40^, mRNA decay^41^, translation regulation^42^, and nuclear export^43^. It follows that METLL3 exhibits its roles in normal human physiological process or diseases by regulating the m6A modification of miRNAs.

Surgery is the frontline treatment for the majority of patients who admit to hospital. Sadly, postoperative death constitutes the third cause of death (7.7% of global deaths per year) with the first and second causes of death being ischemic heart disease and stroke, respectively^44^. Those deaths after surgery involve multiple factors but one key cause is postoperative organ injury^45^. It is not surprising that surgical trauma can cause extensive cell death and then subsequently damage-associated molecular patterns (DAMPs) are released from those dead cells to initiate further biological cascades to cause further cell death and organ injury^46,47^. Arguably, vascular endothelial cell injury is central in all organ injuries and ischemia and reperfusion injury often occurs during and after surgery. Therefore, development of strategies to protect against endothelial cell injury and further alleviate organ injury are very important clinically. Our data clearly demonstrated that propofol inhibited ischemia/reperfusion induced endothelial injury, indicating that its perioperative use may have great potential to protect against endothelial cell/organ injury during and after surgery. However, our study is a pure in vitro study and how this is relevant in an in vivo and/or clinical setting is unknown and warrants further study. Nevertheless, there is clinical evidence showing that general anesthetics protect against organ injuries during and/or after surgery although it has always been debatable. Some papers suggest that volatile anesthesia may produce more myocardial protection compared to propofol^48–51^. Conversely, propofol was also reported to provide comparable myocardial protectivity like that of isoflurane in patients undergoing coronary artery bypass graft surgery^52^. Whilst no difference in myocardial protection with either sevoflurane or desflurane, or total intravenous anesthesia with propofol as assessed by measuring serial cTnT values^53^.

Our work is not without limitations. First, the baseline autophagy in the presence of propofol up to 150 μM without insult was not changed as we reported previously^24^. Whether the protection against H/R-induced autophagic cell death demonstrated in the current study is “insult specific” remains unknown. Second, unlike our data, it has been reported that METTL3 enhances autophagic flux and inhibits apoptosis in H/R-treated cardiomyocytes, suggesting that METTL3 is a negative regulator of autophagy^54^. However, METTL3 is a generic regulator of RNA and the cell type used in our experiments and experimental conditions are different from the previous study; all of which may explain this discrepancy. Finally, for sake of argument, anesthetics can act on any type of body cells and hence the complicated changes of METTL3, miR-20b, and ULK1 under insult and propofol found in this study may be just an association and hence the true targets or mechanistic actions of propofol may be far more than these.

In summary, our findings provide a novel mechanism of METTL3 in preventing H/R-induced autophagic cell death by accelerating pri-miR-20b maturation in a m6A-dependent manner during propofol post-conditioning. In addition, this study elucidated the molecular mechanism of autophagy-related miRNAs in inhibiting excessive autophagy by regulation of the autophagic core protein ULK1, and miR-20b prevented H/R-induced autophagic cell death by regulating ULK1 during propofol post-conditioning.

## Methods

### Cell culture and injurious model

Human umbilical vein endothelial cells (HUVECs) were used in this study. The cells were cultured in DMEM supplemented with 10% fetal bovine serum and 1% penicillin-streptomycin at 37 °C with air containing 5% CO_2_. Hypoxic challenge was achieved with a gas mixture containing 1% O_2_, 5% CO_2_, and 94% N_2_ for 12 h in a hypoxic chamber (Billups-Rothenberg, Del Mar, CA, USA) with or without propofol up to 150 μmol/L for 4 h, and then recovered in the DMEM for further analysis.

### Plasmids, miRNAs, or siRNA transfection

Transfection of plasmids and mimics were performed using Lipofectamine 2000 according to the manufacturer’s instructions. Transient knockdown of target genes was performed with Lipofectamine 3000 and siRNA: MiR-20b mimics (5′-CAAAGUGCUCAUAGUGCAGGUAG-3′; 5′-ACCUGCACUAUGAGCACUUUGUU-3′), negative control (NC) (5′-UUCUCCGAACGUGUCACGUTT-3′; 5′-ACGUGACACGUUCGGAGAATT-3′), miR-20b inhibitor (5′-CUACCUGCACUAUGAGCACUUUG-3′) inhibitor NC (5′-CAGUACUUUUGUGUAGUACAA-3′) or METTL3 siRNA (5′-GGUGACUGCUCUUUCCUUATT-3′;5′-UAAGGAAAGAGCAGUCACCTT-3′) (Genema, shanghai, China). The full-length sequence of ULK1 cDNA was cloned into the LvCGP-C-flag (Longqian Biotech, shanghai, China). The 3′ UTR of ULK1 cDNA was cloned into pmirGLO dual-luciferase miRNA target expression vector (Promega) with XhoI and XbaI restriction sites using the following primers: 5′-TAACTCGAGCCTTTCTGGCCTGGCTGGG-3′ (ULK1-F), 5′-TAGTCTAGATGACACCAGCCCAACAATTCC-3′(ULK1-R). Mutations were made by site-directed mutagenesis, changing microRNA target site from “GCACTTTA” to “cgtgaaat’ or/and “CACTTT” to “gtgaca”(Longqian Biotech, shanghai, China). Q5^®^ High-Fidelity DNA Polymerase (Bio Labs, M0491S) was used for PCR reaction.

### Luciferase assay

HUVECs were seeded in a 12-well plate one day before transfection. When the cell had grown to the density of 70–80%, for reporter assays, they were transiently co-transfected with 0.3 μg of reporter plasmid in the presence of 100 nM NC, miR-20b mimics (20b), inhibitor NC (IN NC), or miR-20b inhibitor (IN 20b) every three wells using Lipofectamine 2000 (this and other reagents are shown in Supplementary Table 1). Firefly and Renilla luciferase activities were measured consecutively by using Dual-Luciferase Reporter Assay System according to the manufacturer’s protocol.

### Quantitative real-time RT-PCR

Total RNA was extracted with TRIzol (Life Technologies, Carlsbad, CA, USA) followed by a DNase treatment to eliminate contaminating genomic DNA (Thermo Fisher Scientific, Boston, MA, USA; B43), and a reverse transcription reaction (Thermo Fisher Scientific; K1622). Amplification and relative quantification of cDNA was carried out with SYBR Premix Ex TaqTM (Tli RNaseH Plus) (TaKaRa, Shiga, Japan; RR420A) according to the manufacturer’s protocol. Relative quantitative PCRs for miRNAs were performed with SYBR PrimeScript miRNA RT-PCR Kit (TaKaRa; RR716). Fold changes were calculated using the 2^−ΔΔCT^ method with normalization to the GAPDH control. The qRT-PCR primers (IBSBIO, Shanghai, China) are shown in Supplemental Table 2.

### Western blot

Proteins used for immunoblotting were collected in lysis buffer containing a phosphatase inhibitor (Roche, Basel, Switzerland; 4693116001) on ice. Lysates were mixed with loading buffer and boiled for 10 min to denature protein. Protein samples were separated by 10% SDS/PAGE and then transferred to a PVDF membrane. Membranes were blocked with 5% non-fat milk dissolved in phosphate buffered saline with tween-20 (PBST) for 1 h at room temperature. Then, the membranes were incubated with various primary antibodies (see Supplementary Table 3) at 4 °C overnight, followed by HRP-labeled secondary antibodies’ incubation at room temperature for 2 h. GAPDH or β-actin antibodies were probed to detect the loading control. Bands’ densitometric data against loading controls were done with ImageJ software (Bethesda, MD, USA).

### Immunofluorescent staining

HUVECs were cultured to reach 70% confluence on coverslips, and then washed with PBS (pre-warmed) and fixed with 4% paraformaldehyde at 37 °C for 20 min after various treatments. The cells were permeabilized with 0.1% Triton X-100 on ice. After blocking in 1% albumin bovine V for 30 min at room temperature, cells were incubated with various primary antibodies (see Supplementary Table 3), diluted in 0.01% Triton X-100 for 1 h at room temperature. Cells were then washed with PBS five times, and then secondary antibodies were applied for another 1 h at room temperature. Cell images were captured with a TCS SPF5 II Leica confocal microscope and software (LAS-AF-Lite_2.2.0_4758; Leica Microsystems, Wetzlar, Germany).

### Electron microscopy

HUVECs were fixed in 2.5% glutaraldehyde in 0.1 M sodium phosphate buffer, pH 7.4, at 37 °C for 2 h, and then dehydrated in a graded ethanol series and embedded. Approximately 70 nm ultrathin sections were mounted on nickel grids. The samples were then stained and visualized using a 120-kV Jeol electron microscope (JEM-1400; JEM, Peabody, MA, USA) at 80 kV. Images were captured using a Gatan-832 digital camera (GATAN, Pleasanton, CA, USA).

### Statistical analyses

Cultured cells in well plates were randomly used in different experimental groups and all experiments were independently carried out at last three times with different sets of cultures. Data are expressed as mean ± SEM and are also presented as dot plot. Statistical analyses were performed using one-way ANOVA with Bonferroni’s multiple comparisons (GraphPad Software Inc., San Diego, CA, USA). A *p* value < 0.05 was considered statistically significant.

## Supplementary information

online supplements
