# Supplementary material for: Propofol-induced MiR-20b expression initiates endogenous cellular signal changes mitigating hypoxia/re-oxygenation-induced endothelial autophagy in vitro
Source: Cell Death Dis. 2020 Aug 13;11(8):681. doi: 10.1038/s41419-020-02828-9 (PMC7442825; doi:10.1038/s41419-020-02828-9)
Supplement: Supplementary file 1 — online supplements [file 41419_2020_2828_MOESM1_ESM.pdf]

Online supplements

Supplemental table 1. Reagents were used in the experiments

Reagents and materials	Product Code	Supplier
Propofol	D126608	Sigma -Aldrich
Dual-Luciferase® Reporter Assay System	E1910	Promega
polyvinylidene fluoride (PVDF) membranes	ISEQ00010	Millipore
Albumin Bovine V	A8020	Solarbio
Lipofectamine 2000	11668027	Invitrogen
Lipofectamine 3000	L3000008	Invitrogen
TransScript One-Step gDNA Removal and cDNA Synthesis SuperMix	AT311-03	TransGen Biotech
Dulbecco’s modified Eagle’s medium (DMEM)	C11965500BT	Gibco
fetal bovine serum	16000044	Gibco

Supplemental table 2. Primers were used for qRT-PCR

Primer	sequence
$U6^F$	5’-CTCGCTTCGGCAGCACA-3’
$U6^R$	5’-AACGCTTCACGAATTTGCGT-3’
$GAPDH^F$	5’CCACCCAGAAGACTGTGGAT-3’
$GAPDH^R$	5’CACATTGGGGGTAGGAACAC-3’
Hsa-miR-20b-$5P^F$	5’ATGGTTCGTGGGCAAAGTGCTCATAGTGCAGGTAG-3’
Hsa-miR-20b-$5P^R$	5’CTCAACTGGTGTCGTGGA-3’
$ULK1^F$	5’GGTCACACGCCACATAACAG-3’
$ULK1^R$	5’TGAGAATAAAGCCATCAAGGTG-3’
$Beclin1^F$	5’GAAGACACAGGAGGCAGTGG-3’
$Beclin1^R$	5’AGGACACCCAAGCAAGACC-3’
pri-miR-$20b^F$	5’AGAGGATAAGATTGGGTCCTA-3’
pri-miR-$20b^R$	5’ACAAGAGATTTGTTATCCAAGA-3’
$METTL3^F$	5’ACACTGCTTGGTTGGTGTCA-3’
$METTL3^R$	5’AATCTTTCGAGTGCCAGGGG-3’

Supplemental table 3. Antibodies were used in the experiments

Primary Antibodies	Product Code	Supplier	Application
anti-Atg1/ULK1 antibody rabbit mAb	A7481	Sigma -Aldrich	WB
anti-ULK1 antibody	ab128859	Abcam	WB、IF
anti-beclin1 antibody	ab114071	Abcam	WB
anti-P62 mouse mAb	ab56416	Abcam	WB、IF
anti-LC3 antibody	ab51520	Abcam	WB、IF
anti-LC3	L7543	Sigma -Aldrich	WB
anti-beta-Actin antibody	sc-47778	Santa Cruz	WB
anti-Flag	ab1257	Abcam	WB
anti-METTL3 antibody	ab195352	Abcam	WB
anti-DGCR8 antibody	ab191875	Abcam	WB
anti-GADPH Mouse Monoclonal antibody	HC301-01	TransGen Biotech	WB
anti-TOM20 (FL-145)	sc-11415	Santa Cruz	IF
Secondary Antibodies	Product Code	Supplier	Application
HRP affinipure goat anti-rabbit IgG	E030120	Earthox	WB
HRP affinipure goat anti-mouse IgG	E030110	Earthox	WB
Alexa Fluor 488-labeled donkey anti-mouse IgG	A21202	Invitrogen	IF
Alexa Fluor 488-labeled donkey anti-rabbit IgG	A21206	Invitrogen	IF
Alexa Fluor 555-labeled donkey anti-mouse IgG	A31570	Invitrogen	IF
Alexa Fluor 555-labeled donkey anti-rabbit IgG	A31572	Invitrogen	IF
Alexa Fluor 647-labeled donkey anti- mouse IgG	A32787	Invitrogen	IF
Alexa Fluor 647-labeled donkey anti-rabbit IgG	A32733	Invitrogen	IF
